# BuffExDb: web-based tissue-specific gene expression resource for breeding and conservation programmes in *Bubalus bubalis*

**DOI:** 10.1093/database/baae128

**Published:** 2025-01-24

**Authors:** Naina Kumari, Samir Kumar, Anupama Roy, Princy Saini, Sarika Jaiswal, Mir Asif Iquebal, Ulavappa B Angadi, Dinesh Kumar

**Affiliations:** Division of Agricultural Bioinformatics, ICAR-Indian Agricultural Statistics Research Institute, Library Avenue, PUSA, New Delhi 110012, India; The Graduate School, ICAR-Indian Agricultural Research Institute, Library Avenue, PUSA, New Delhi 110012, India; Cochin University of Science and Technology, Kerela, Kochin 682022, India; Division of Agricultural Bioinformatics, ICAR-Indian Agricultural Statistics Research Institute, Library Avenue, PUSA, New Delhi 110012, India; Division of Agricultural Bioinformatics, ICAR-Indian Agricultural Statistics Research Institute, Library Avenue, PUSA, New Delhi 110012, India; Division of Agricultural Bioinformatics, ICAR-Indian Agricultural Statistics Research Institute, Library Avenue, PUSA, New Delhi 110012, India; Division of Agricultural Bioinformatics, ICAR-Indian Agricultural Statistics Research Institute, Library Avenue, PUSA, New Delhi 110012, India; Division of Agricultural Bioinformatics, ICAR-Indian Agricultural Statistics Research Institute, Library Avenue, PUSA, New Delhi 110012, India; Division of Agricultural Bioinformatics, ICAR-Indian Agricultural Statistics Research Institute, Library Avenue, PUSA, New Delhi 110012, India

## Abstract

Amidst the global challenge of extreme poverty, the livestock sector can significantly contribute to global sustainable development goals by enhancing resilience, smallholder productivity, and market participation. The Indian livestock sector is one of the largest in the world with a total livestock population of 535.82 million, ∼10.7% of the world’s livestock population. Buffalo (*Bubalus bubalis*) holds significant importance in India and other Asian countries, notably contributing to their economies by surpassing cattle in milk production and providing various valuable products. The limited availability of genomic and transcriptomic resources for buffaloes hinders the efforts to enhance their traits for increased milk and meat production. To address this gap, this study adopted the state-of-the-art bioinformatics tools to analyse 2429 transcriptomes representing 438 BioSamples from 23 BioProjects obtained from a public domain database, representing 76 different types of tissues and cell types from all major organ systems in buffalo species (river and swamp). The outcome of this exhaustive genomic data led to the development of a relational buffalo expression database based on a three-tier architecture named as BuffExDb (http://46.202.167.198/buffex/). The user-friendliness and flexibilities in retrieval of tissue-specific genes (TSGs) and their functional annotation are the major characteristics of BuffExDb. This is the first of its kind that offers an effortlessly navigable and filterable database, enabling users to examine and visualize the expression levels of each tissue across multiple samples, simultaneously. It also provides the Tau score parameter for the identification of TSGs along with their essential roles in tissue development, maintenance, and function as observed through the enrichment test for gene ontologies. The exhaustive outcome of this work would pave the way for the biological, functional, and evolutionary studies for easy access. This prior information based on tissue-specific mechanisms can be used for future genomic research, especially in association studies in endeavour of enhanced buffalo breeding and conservation programmes.

**Database URL**: http://46.202.167.198/buffex/

## Introduction

The major global challenges include extreme poverty, starvation, unemployment, degradation of the environment including soils, oceans, and forests, and loss of biodiversity [1]. The livestock sector can play a key role in addressing these challenges by contributing to improved productivity of smallholders, increasing their resilience and participation in markets, thus also enabling the achievement of sustainable development goals [2]. Livestock products are known to supply up to 18% of the global calories and 40% of proteins in the human diet globally while also providing essential micronutrients such as vitamin A, vitamin B-12, riboflavin, calcium, iron, and zinc, which are most important for combating hidden hunger and enhancing food security [3]. Livestock production can improve water quality and nutrient recycling, mitigating public health risks and supporting biodiversity. Manure management and energy recovery from animal waste, for instance through anaerobic digestion, can offer sustainable energy options, thereby contributing to affordable and clean energy [4, 5]. Major livestock species include cattle, buffalo, sheep, goats, pigs, mithun, and yak [6]. The domestic water buffalo (*Bubalus bubalis*), falls under the family Bovidae and the subfamily Bovinae, also referred to as the wild Indian buffalo. There are two distinct classifications for water buffalo, namely swamp buffalo and river buffalo. The swamp buffalo is prevalent in Southeast Asia and is primarily used as a draught animal. On the other hand, the river buffalo is the predominant type in countries such as India, Pakistan, Bulgaria, Hungary, Turkey, Italy, Egypt, and Brazil. River buffaloes have been primarily bred for enhanced milk production [7].

Global bovine milk production has raised by 58% to 884 million tonnes in 2021, which is primarily obtained from cattle and buffalo, accounting for 96% of total milk output. The Indian livestock sector is one of the largest in the world with a total livestock population of 535.78 million (10.7%), of which the buffalo population accounts for 20.5% (109.85 million) as recorded by the 20th livestock census of India, 2019 [6]. India accounts for 23% of global milk production and contributes significantly to global meat production (2.42%), where cattle and buffalo are the major contributors. However, global milk production is also driven by various other countries, including Pakistan (11%), China (7%), Brazil, Germany, the Russian Federation, and France (4%) [8]. The Committee on Doubling Farmers’ Income (2018) identified the sectors of dairying, cattle, poultry, fisheries, and horticulture as critical industries, emphasizing their expansion. With over eight crore farmers involved, the dairy sector holds significance in agricultural production [9]. According to the Central Coastal Agricultural Research Institute, buffalo farming holds significant importance within the livestock industry, providing over 50 million tons of milk, 1.43 million tons of meat, and valuable by-products such as hides and bones [10]. Additionally, buffaloes contribute to agricultural operations through their use as draft power.

In this era of genomics, the advent of low-cost sequencing machines has given rise to an ample amount of data generation. Subsequently, RNA sequencing (RNA-Seq) has become a powerful technology that allows the investigation of genome-wide gene expression. Researchers can get valuable insights about the functioning of genes by examining transcriptome data [11–13]. Moreover, gaining insight into the transcriptome is crucial for deciphering the genome’s functional components, uncovering the molecular makeup of cells and tissues, and comprehending growth and illness. It is feasible to compare experiment findings directly and ascertain the absolute amount of each molecule in a cell population using RNA-Seq [14]. Moreover, gene expression profiling can help in multiple areas of drug discovery such as target identification, pharmacogenomics, biomarker development, compound selection, and toxicology by elucidating the engagement of specific cellular pathways in specific organs or tissues [15]. Gene expression data can be helpful in understanding genetic plasticity that facilitates the identification of genes associated with traits such as heat tolerance, disease resistance, and feed efficiency. This can help develop breeding strategies aimed at enhancing resilience to environmental stressors, thereby improving productivity and sustainability in livestock systems [16]. Recent advancements in genome-editing techniques [17] provide additional opportunities for enhancing gene expression profiles in farm animals. These techniques enable precise modifications that can improve specific traits in *B. bubalis* species. Furthermore, studies have shown that multi-tissue global gene expression analyses can reveal regulatory signatures that are critical for improving important traits in cattle [18]. Similar methodologies can be adopted to identify gene signatures pertaining to important traits in buffalo offering insights into metabolic processes that are vital for their management and breeding.

Additionally, various transcriptome resources in the form of databases and web servers, made possible by technologies such as RNA-Seq, are priceless tools that offer deep insights into the dynamics of gene expression and molecular biology processes, which are crucial for comprehending a wide range of biological phenomena. There are relatively few expression atlases on bovine species, many of which focus on the cattle (*Bos taurus*) species, such as *The Bovine Gene Atlas* [19], *The Cattle Gene Atlas* [20], *CattleGTEx* [21], and *EMBL-EBI expression atlas* [22]. Transcriptome databases for buffalo are relatively scarce due to the limited availability of genomic resources for this species. The first draft quality reference sequence for river buffalo was obtained from a female Italian Mediterranean buffalo (Olimpia) breed, which was assembled at the chromosome level (GCA_000471725.1), submitted to the National Center for Biotechnology Information (NCBI) in 2013 [23]. The NCBI database lists a total of seven genome assemblies for the domestic water buffalo, where four genome assemblies are available at the scaffold level, two are present at the chromosome level, and only one complete genome (NDDB_SH_1) from the Murrah breed, submitted in 2019. It has been assigned as the RefSeq (Reference Sequence) genome by the NCBI, USA [24]. However, so far there is no published genome sequence available for the swamp buffalo.

Amidst the scanty information and research on buffalo, there is a need for a user-friendly, comprehensive, and exhaustive web genomic resource in the form of atlas for tissue-wise gene expression. In a previous study, gene expression data for 50 tissues and cell types, comprising approximately 220 biosamples, were presented [25]. This research was expanded by generating gene expression information for 19 tissues from swamp buffaloes and 51 tissue types from river buffaloes later in 2023 [26]. However, neither of these previously developed expression atlases provided a comprehensive and user-friendly gene expression database suitable for the broader community. Our present study aims at analysing RNA-Seq data from buffalo species available to date, utilizing 438 BioSamples encompassing 76 tissues and cell types obtained from publicly available databases. This study also aims to facilitate easy access and utilization of tissue-wise expression datasets of buffalo in the form of a user-friendly buffalo expression database, BuffExDb for the research community. This rich and diverse dataset will provide a valuable resource for conducting comprehensive analyses of gene expression patterns across various tissues and cell types in buffalo. This will aid the bovine researchers to have a better insight into the tissue-specific molecular mechanism in the complex traits and diseases, facilitating future genome-wide association studies. The findings of this work are catalogued in the form of a user-friendly web-genomic resource of tissue-based buffalo transcriptome that would pave the way for the biological, functional, and evolutionary studies in endeavour of livestock improvement and management.

## Materials and methods

### Data collection and preprocessing

RNA-Seq data were downloaded from the NCBI [27] using the ‘Sequence Read Archive toolkit’. The dataset was extensive, encompassing 2483 RNA-Seq datasets, derived from 29 BioProjects, involving 468 BioSamples. The diversity of the dataset is reflected in the inclusion of both paired-end and single-end datasets, covering 76 different tissues and cell types from the river buffalo (*B. bubalis*) and swamp buffalo (*B. bubalis carabanensis/kerabau*) (Supplementary Table S1).

The downloaded raw fastq files were checked for the quality of sequence reads, presence of adapters, Guanine-Cystosine content, and presence of *k*-mers using the ‘FASTQC’ [28] tool to assess the sequencing errors. Paired-end samples with an unequal number of sequences in the pairs were excluded from subsequent analysis. ‘Trimmomatic’ v0.39 was then employed to trim adapters and low-quality bases, with a minimum length threshold set at 36 [29]. Samples in which >60% of reads were discarded during trimming were excluded from further analysis. The parameters used for trimmomatic were TrueSeq3-SE.fa/TrueSeq3-PE.fa:2:30:10 LEADING:10 TRAILING:10 SLIDINGWINDOW:4:20 HEADCROP:13 MINLEN:36. The given settings indicates removal of adapters sequences with seed matches of 16 bases, allowing maximum 2 mismatches. Adapter sequences were extended and clipped if paired end reads had a score of 30 or single end reads had a score of 10. Additionally, leading and trailing low quality bases or ‘N’ bases (below quality 10) were removed. Reads were scanned using a 4-base wide sliding window, cutting them when the average quality per base drops below 20.

### Alignment to the reference genome

Indexing of the buffalo reference genome and alignment of cleaned reads to the reference genome NDDB_SH_1 (GenBank ID: GCA_019923935.1) were performed using the ‘HISAT2’ (v2.2.1) [30] tool. Extraction of splice sites and exons was performed before indexing the reference genome, which was then used to index the reference genome. The samples with unique mapping rates ≥90% were kept for downstream analysis. ‘Samtools’ v9.1 was used to convert sam to bam file formats [31].

### Transcript assembly and quantification of expression level

**Figure 1.**
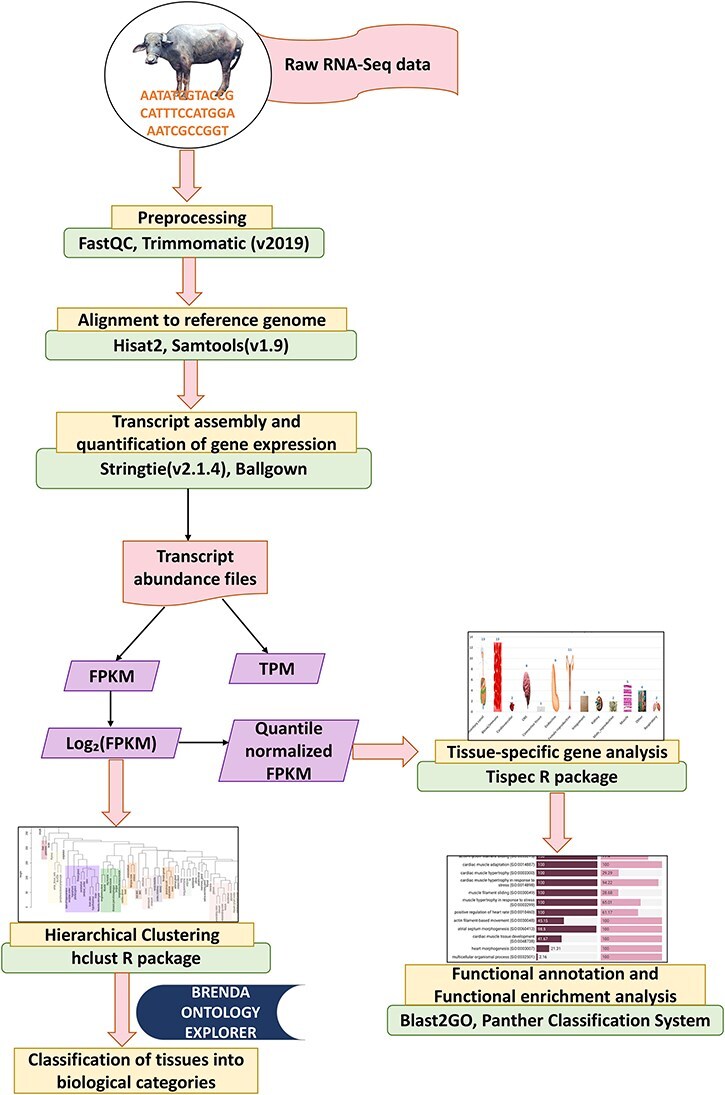
Analysis pipeline for RNA-Seq data.

‘Stringtie’ (v2.1.4) [32] tool was employed for assembly of the transcripts in each dataset. It estimates the expression level of each gene and transcript as it assembles them. Following the assembly of every sample, merge function of ‘StringTie’ was used to receive the whole set of assemblies and to combine all gene structures present in any of the samples. In order to compare the transcripts in later phases, the merge step produces a set of transcripts that are consistent over all samples. The transcript abundances in FPKM (Fragments Per Kilobase of transcript per Million mapped reads) and TPM (transcript per million) were then calculated in each sample using the merged gene structures while accounting for sequence depth and gene length differences across samples [33]. Prediction of coding and noncoding genes was performed using ‘CPC2’ [34] tool. Figure 1 illustrates the RNA-Seq analysis pipeline for analysing the RNA-Seq data in this study.

### Hierarchical clustering and tissue classification into biological categories

The mean FPKM values were calculated for each tissue and cell type and subsequently subjected to logarithmic transformation (log2) [20]. Hierarchical clustering of all tissues was conducted using the ‘hclust’ function from the ‘dplyr’ library in R (v4.1.2) [35, 36]. The clustering was based on the ‘Euclidean’ distance matrix and the ‘average’ method [37].

With the help of BRENDA, the tissue ontology explorer tool [38], the 76 types of tissues were categorized into 13 different categories of tissues [19, 20]. The BRENDA Tissue Ontology (BTO) is an extensive, structured encyclopaedia that offers definitions, classifications, and terms for organs, tissues, cell cultures, types of cells, anatomical structures, plant parts, and organisms from all taxonomic groups—including fungi, animals, and plants in accordance with the guidelines and conventions of the Gene Ontology (GO) [39].

### Identification of tissue-specific genes and their functional enrichment analysis

To identify genes specific to the 76 types of tissues and cells, tissue enrichment analysis was performed using tau ($\tau $) index from the ‘tispec’ v0.99 package in ‘R’ (v4.1.2) [40, 41] to obtain absolute-specific, highly specific, intermediate-specific, and housekeeping genes. The tau index is reported to give the genes that are evolutionarily conserved tissue specific [42]. FPKM values for all individual samples were used to estimate the gene expression level for each tissue. The FPKM values were log-transformed with base 2 and quantile-normalized before performing the tissue-specific gene (TSG) analysis. The tau index ranges from 0 to 1, where genes with a tau index of 1 were categorized as absolute TSGs. The tau index between 0.82 and 1 is categorized as highly specific, between 0.2 and 0.8 as intermediate specific, and below 0.2 as nonspecific/housekeeping gene.

The functional annotation of the identified genes was performed using the ‘BLAST2GO’ tool [43]. GO-slim analysis was employed to obtain the gene ontologies for molecular, biological, and cellular functions across all tissues and genes. Identification of pathways was performed using the Kyoto Encyclopedia of Genes and Genomes (KEGG) pathway [44] from the ‘BLAST2GO’ function. Functional enrichment analysis for TSGs was performed using the ‘PANTHER (v14.0) classification system’ [45] to obtain enriched GO biological process, molecular function, and cellular component, which was followed by enrichment analysis using *Bos taurus* as the reference organism. This was done due to the conservation of genes between these two closely related bovine species [26]. For statistical analysis, Fisher’s exact test along with a false discovery rate (<0.05) was performed to obtain the enriched gene ontologies for each tissue.

### Development of comprehensive tissue-wise expression atlas of buffalo

**Figure 2.**
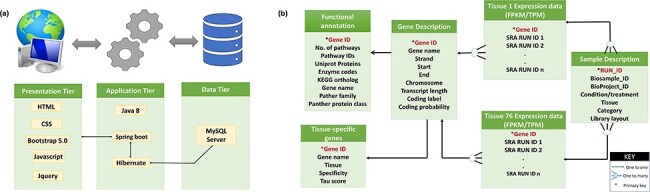
(a) Three-tier structure of the database. (b) Tables in the database and their relations.

A user-friendly tissue-wise expression atlas of buffalo species was developed to facilitate the users with the expression values for each tissue in each sample. This expression database is a relational database constructed using a three-tier architecture. For the front end, i.e. the presentation layer (client-tier) development, CSS, HTML, Bootstrap 5.0, JavaScript, and JQuery (JavaScript Library) were employed. The application layer (logical tier) was built on the Java 8 language using Spring Boot and Hibernate as the frameworks. The data tier (database) is built using the MySQL server. The database includes 76 tables for each tissue showing its expression in multiple samples measured in FPKM and TPM. Additionally, it contains four other tables for sample description, gene description, TSGs, and functional annotation. Figure 2a illustrates the database structure, and Fig. 2b illustrates the relationships between the tables.

## Results and discussion

### Summary metrics of the RNA-Seq dataset

The dataset included in the study comprises 2483 RNA-Seq data from 29 different BioProjects of buffalo transcriptome available in the NCBI. After preprocessing of the raw reads, 2429 RNA-Seq data were proceeded for further analysis, while the rest were dropped due to a smaller number of reads retained after trimming. Finally, 23 and 438 BioProjects and BioSamples, respectively, were retained post-trimming. On average, 5546867 clean reads were obtained from 2429 datasets with a mapping rate of > 90%. The normalized expression levels in the form of FPKM and TPM for 37 164 transcripts were obtained. Analysis of coding potential using the ‘CPC2’ tool identified 16 529 genes as coding with a coding probability of >.5. An average number of 23 868 genes (median = 22 888) was expressed in all tissues with FPKM > 0 ranging from 17 040 to 34 742 genes in popliteal lymph nodes and adipose tissues, respectively. According to the Human Protein Atlas, the human tissue-specific proteome shows that 69% of the total protein in humans is expressed in the adipose tissue [46].

### Hierarchical clustering and tissue classification into biological categories

**Figure 3.**
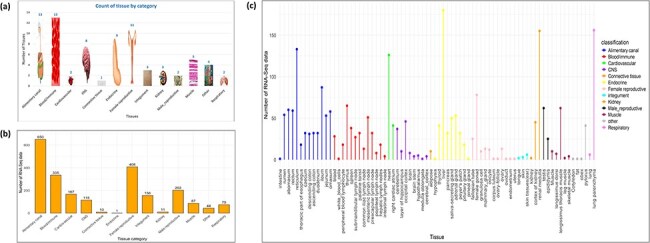
(a) Distribution of RNA-Seq data across various tissue categories, with bar heights indicating the number of RNA-Seq datasets for each category. (b) The number of tissues per category, with images scaled according to the tissue count. (c) RNA-Seq data distribution across individual tissues, with bar colours representing their respective tissue categories.

**Figure 4.**
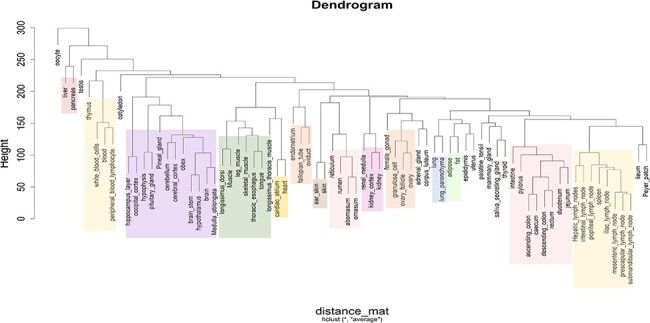
Dendrogram showing the hierarchical clustering of tissues based on gene expression (log2 FPKM) data. The different colours in the clusters represent different tissue categories.

Based on the classification of tissues and cell types from the BRENDA tissue ontology (BTO) explorer [38], 13 categories were obtained for the 76 types of tissues, namely alimentary canal, blood/immune, cardiovascular, central nervous system (CNS), connective tissue, endocrine, female reproductive, integument, kidney, male reproductive, muscle, respiratory, and other. The tissues that could not be found in BTO were kept in the ‘other’ category. Figure 3a shows the number of tissues in each category, Fig. 3b shows the number of RNA-Seq data in each category, and Fig. 3(c) shows the category of each tissue along with the number of RNA-Seq data in each tissue type. Hierarchical clustering of all tissues and cell types was performed based on the log normalized gene expression levels. Figure 4 represents a dendrogram using the ‘hclust’ and ‘ggplot2’ packages in R (v4.1.2). Tissues of the blood/immune category were observed in two clusters where all lymph nodes and spleen form a single cluster and white blood cells (WBCs), blood, peripheric blood lymphocytes, and thymus form another cluster. All tissues of the CNS category, namely layer of hippocampus, occipital cortex, cerebellum, cerebral cortex, brain stem, pineal gland, hypothalamus, brain, medulla oblongata, obex, and pituitary gland form one large clade. Based on BTO, pineal gland and pituitary gland can be classified in both the categories of endocrine and CNS. Nonetheless, based on the hierarchical clustering, pineal gland and pituitary gland fall in the CNS cluster. Similarly, tissues of muscle category (longissimus dorsi, longissimus thoracis, leg muscle, tongue, and skeletal muscle) form one cluster. The results of hierarchical clustering are in accordance with the classification of tissues into biological categories by BTO. The dendrogram in Fig. 4 shows the clustering of tissues belonging to the same category cluster together, despite samples belonging to different experimental conditions [20, 37].

### Functional annotation and identification of TSGs and their functional enrichment analysis

**Figure 5.**
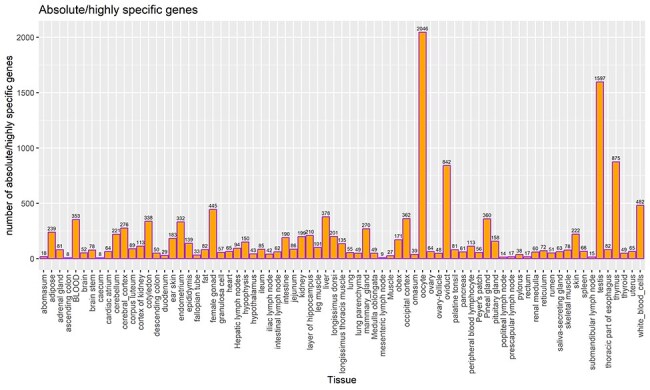
Count of TSGs (including both highly specific and absolute specific genes) for each tissue. The bar label indicates the total number of highly specific genes and absolute TSGs.

TSGs were identified based on the tau score for each gene and categorized as described in the method section. In total, 14 111 and 9662 genes were categorized as highly/absolute specific (tau score >0.8) and intermediate specific (0.2 > tau score < 0.8), respectively, across all tissues and cell types. A total of 2263 genes (tau score <0.2) were categorized as housekeeping genes, i.e. present across all tissues. Among all tissues, oocyte shows the highest number (2046) of absolute/highly specific genes followed by testis (1597) and thymus (875) tissues. Previous studies report the testis tissues to have the highest number of TSGs in cattle [20, 26], as well as in humans [47]. The least number of absolute/highly specific tissues were detected in ascending colon and caecum, i.e. eight in each. Figure 5 lists the number of TSGs in each tissue, including both absolute and highly specific genes.

Statistical overrepresentation test for TSGs (absolute + highly specific) of GO terms indicated that tissues belonging to the same category showed similar GO terms enriched within them. This suggests that the functional roles of these tissues are closely related and that their TSGs are involved in similar biological processes. For Instance, in case of GO terms related to biological processes, TSGs from the blood/immune system, namely blood, white blood cells, and peripheral blood lymphocyte, were significantly enriched in GO terms such as cytokine-mediated signalling pathway (GO:0019221), immune response (GO:0006955), immune system process (GO:0002376), inflammatory response (GO:0006954), and leukocyte activation (GO:0045321). These GO terms are consistent with the functions of the blood/immune system as they are related to the immune system’s response to foreign molecules and the activation of leukocytes. Genes coding for pro-inflammatory cytokines such as IL1, IL1B, IL23R, IL6, and toll-like receptor proteins such as TLR2 and TLR8 were found to be absolute/highly specific in the blood/immune system. The results are consistent with the gene cluster of the immune system (Cluster 19) obtained by Young *et al*. [25]. Additional genes identified within this category include *OASL* (2ʹ-5ʹ-oligoadenylate synthetase like), which plays a crucial role in defending against viral infections. Additionally, *CXCL8* (C-X-C motif chemokine ligand 8) and *CXCR2* (C-X-C motif chemokine receptor 2) are involved in orchestrating the accumulation and activation of white blood cells at inflammatory sites. Research indicates that single-nucleotide polymorphisms within *CXCL8* and *CXCR2* could serve as potential genetic indicators for enhancing udder health in Simmental buffalo breeds [48]. The *OASL* gene is found to be linked with the dry matter intake trait in cattle. Blood is the major source for the absorption and transportation of nutrients and metabolites to different organs and tissues. Blood metabolites play a direct role in metabolic processes as substrates or products, positioning them as promising subjects for further investigation into feed efficiency [49].

**Figure 6.**
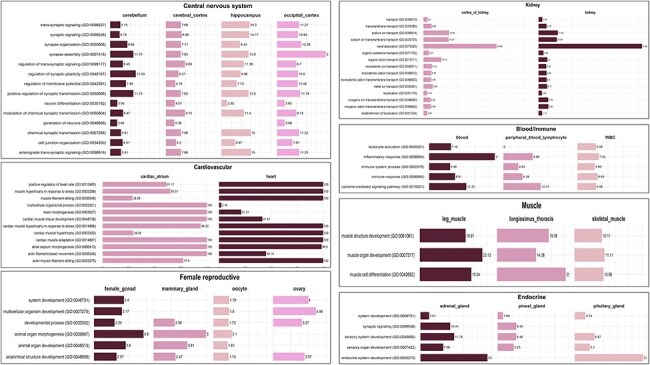
Common enriched GO (biological process) terms for tissue categories: CNS, cardiovascular, kidney, endocrine, muscle, and female reproductive. The values in the bar represent fold enrichment for each enriched GO term in each tissue (*P*-value < .05).

The biological processes enriched in tissues from the CNS such as cerebellum, cerebral cortex, hippocampus, and occipital cortex were associated with nervous system development (GO:0007399), chemical synaptic transmission (GO:0007268), neuron differentiation (GO:0030182), synaptic signalling (GO:0099536), and others. These GO terms are associated with the development and operation of the nervous system, aligning with the functions attributed to the CNS. Some genes such as *HOMER1, MAGI2, NRG3*, *CTNNA2*, and *ADGRL3* were found to be highly specific in the occipital cortex. It has been reported that these genes were found to be associated with regulation of neurons and their differentiation and axonogenesis in Fuzhong Buffalo [50]. Studies have shown that the *HOMER1* gene is involved in the development of the nervous system in swamp buffalo and plays an important role in their behaviour [51]. Tissues from the endocrine system, specifically adrenal gland, pineal gland, and pituitary gland, show involvement in similar biological processes such as sensory organ development (GO:0007423), sensory system development (GO:0048880), synaptic signalling (GO:0099536), and system development (GO:0048731). The pineal and pituitary glands are neuroendocrine and are reported to play a vital role in the development of sensory organs in the vertebrate head, specifically the eye [52]. These GO terms are related to the development and functioning of the endocrine system, which is consistent with the functions of these tissues. Tissues from the muscle category, namely leg muscle, skeletal muscle, and longissimus thoracis, show three common biological processes: muscle organ development (GO:0007517), muscle cell differentiation (GO:0042692), and muscle structure development (GO:0061061), reflecting their roles in the growth, development, and functioning of muscle tissues. The common biological processes among female reproductive tissues, namely female gonad, mammary gland, oocyte, and ovary, are anatomical structure development (GO:0048856) and developmental process (GO:0032502). The endometrium and corpus luteum tissues did not show any common biological process with other female reproductive tissues. A similar case has been reported in cattle where the mammary gland tissues were found to be negatively correlated with corpus luteum and endometrium tissues highlighting the antagonistic relationship between fertility and milk yield in animals [20, 53]. Tissues of kidney and kidney cortex share ∼39 biological processes, some of which include renal absorption (GO:0070293), monoatomic anion homeostasis (GO:0055081), chloride ion homeostasis (GO:0055064), sodium ion homeostasis (GO:0055078), and kidney epithelium development (GO:0072073). These biological processes are important for the proper functioning of the kidneys and for maintaining the body’s fluid and electrolyte balance [54]. Nephron development (GO:0072006), metanephros development (GO:0001656), and nephron tubule development (GO:0072080) are also some of the enriched biological processes in kidney tissues, which are related to the development of nephrons, the functional units of kidney. The tissues of the group alimentary canal did not show any common overrepresented biological process among them. Figure 6 delineates the graphical presentation of the common biological processes in all tissue categories.

**Figure 7.**
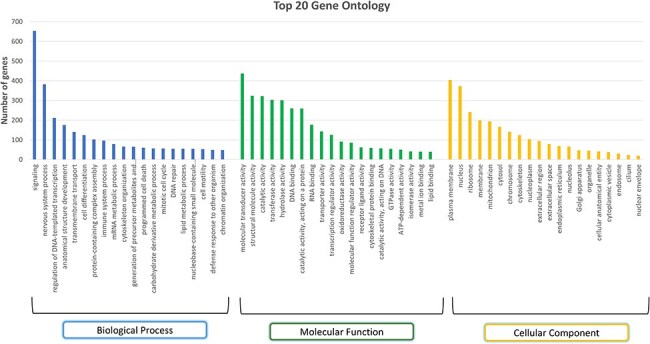
Top 20 Gene Ontology (GO) terms in each category: biological process, molecular function, and cellular component.

A total of 394 pathways were identified using the KEGG pathway database that mapped to 3511 gene sequences, and a total of 4032 pathways were identified from the Reactome pathway that mapped to 10 401 gene sequences. GO analysis identified 110 biological processes, 67 molecular functions, and 17 cellular components across all genes. The top three abundant biological processes observed were signalling (GO:0023052), nervous system process (GO:0050877), and regulation of DNA-templated transcription (GO:0006355). Among the 653 genes that mapped to the ‘signalling’ biological process, the *S1PR1* gene is of particular interest as it has been identified as the candidate gene for marbling of meat, an important economic trait in buffalo [55]. Around 382 genes were seen to be mapped to the nervous system process, one of which is *ADCY5* (Adenylate cyclase 5), which is found to be associated with ovarian morphological-related traits in bovine animals [56]. The top three abundant molecular functions were molecular transducer activity (GO:0060089), structural molecule activity (GO:0005198), and catalytic activity (GO:0140096; GO:0003824). These molecular functions mapped to 437, 324, and 322 genes, respectively. The molecular transducer activity (GO:0005198) was one of the top molecular functions responsible for high altitude adaptation as reported in Ladakhi cows [57]. Plasma membrane (GO:0005886), nucleus (GO:0005634), and ribosome (GO:0005840) are the three most abundant cellular components. Figure 7 represents the top 20 GO terms for each category: biological processes, molecular functions, and cellular components. The PANTHER system identified 10 073 protein families, derived from a single gene duplication event with a common ancestor, and 10 561 protein classes, which group these families based on functional and evolutionary relationships [58]. For instance, genes such as *CSN2*, *CSN3*, and *CSN1S1* were classified into the same protein class, i.e. ‘storage protein (PC00210)’. These genes were found to show high expression during late lactation phases in Murrah buffalo [59]. They were classified into ‘BETA-CASEIN (PTHR11500:SF0)’, KAPPA-CASEIN (PTHR11470:SF2), and ALPHA-S1-CASEIN (PTHR10240:SF0) panther families. All three proteins in our data show the highest expression in the mammary gland tissue. Thus, such annotations can help us in relating genes to their important traits in the buffalo species. Genes related to heat shock proteins (HSPs) such as *HSPA13*, *HSPA2*, *HSPA9*, *HSPA8*, and *HSPA4* were classified into ‘Hsp70 family chaperone (PC00027)’, and *HSP90AB1*, *HSP90B1*, and *HSP90AA1* were classified as ‘Hsp90 family chaperone (PC00028)’ protein class. HSPs are essential for the maturation, refolding, and breakdown of proteins. In addition to aiding in the development of thermotolerance in agricultural animals like cattle and buffaloes, HSPs may also be used as biological markers to gauge the severity of heat stress in livestock [60, 61].

### Development of comprehensive tissue-wise expression atlas of buffalo, BuffExDb

**Figure 8.**
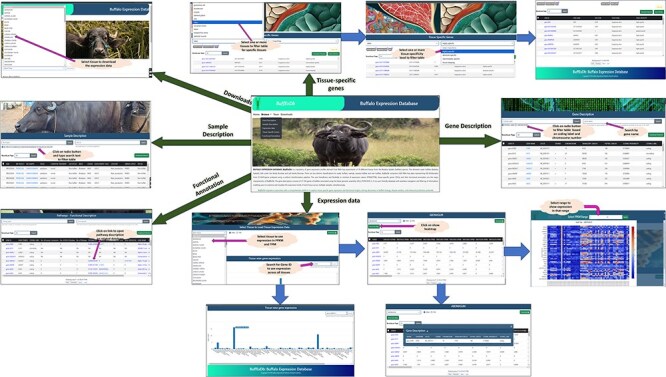
Interface of BuffExDb. The home page comprises four tabs: ‘Home’, ‘Browse’, ‘Team’, and ‘Downloads’. The ‘Browse’ page consists of five drop-down menus: gene description, sample description, expression data, tissue-specific genes, and functional classification. Arrows indicate navigation flow, with some leading to pages accessible from the home page and others pointing to search results generated within each section.

The comprehensive tissue-wise Buffalo expression database, abbreviated as BuffExDb, is a relational database developed using the ‘three-tier architecture’ (http://46.202.167.198/buffex/). It comprises five sections in the BROWSE tab, namely Gene description, Sample description, Expression data, Tissue-specific genes, and Functional annotation. The ‘gene description’ page consists of the list of gene symbols, their start and end positions, locus, strand, chromosome, transcript length, peptide length, coding probability, and coding label. This page can be explored by gene name and chromosome number or by coding label. The ‘sample description’ page lists the BioProject IDs, Run IDs, BioSample, Tissue name, Category of tissue, Condition/Treatment, species, breed, gender, development, age, and library layout. This page can be filtered by BioProject ID, BioSample ID, breed name, condition/treatment, and tissue name or tissue category. The description of a single Run ID can be accessed via a separate search box. The ‘expression’ page shows the expression of each Run ID in FPKM and TPM. The user needs to select any of the tissues from the drop-down menu to see the expression in FPKM, TPM, or both. The default expression is opened in the FPKM format. The user can click on the gene name and sample ID to view its description in a pop-up box from the expression table. The heat map can be viewed by clicking on the show heat map button and by selecting a range of FPKM values. The user can also hover over the heat map to see the value of a particular sample in a particular gene. The heat map can be downloaded in .png format through the button provided in the popup box. In the last section of the page, the user can visualize the expression of a particular gene across all tissues depicted in the bar graph. The TSG page lists the genes identified as tissue specific, which are categorized as absolute specific, highly specific, and intermediate specific. The user can filter the table by selecting the tissue name and any of the tissue-specific categories to obtain a list of the genes in each category along with its tau score for each gene. The functional annotation page describes the KEGG pathway IDs, UniProt protein IDs, enzyme codes, and KEGG ortholog IDs as annotated by the ‘BLAST2GO’ tool, along with the gene name, panther family/subfamily, and panther protein class as annotated by the PANTHER classification system for the genes annotated by the two software. All pages have a download function available. The user can click on the checkboxes available for each row to download specific rows in a text file or click on the top checkbox to download all rows of a specific table. Moreover, a download page is available separately to download the gene expression table for each tissue from the given drop-down menu. Additionally, download options for full tables from gene description, sample description, TSGs, and functional annotation can also be obtained from here. Figure 8 shows the interface of the database and its various pages and interactive options available throughout the webpage.

The previous research in the field of buffalo expression shows the first atlas to be developed for the domestic water buffalo, comprising 57 tissues and 220 BioSamples from the Mediterranean water buffalo breed [25]. Expression atlas on water and swamp buffalo was generated, consisting of 50 tissue types and 355 BioSamples in total [26]. Both the atlases share information on the expression profiles and TSGs in buffalo species but lack a comprehensive user-friendly interface in the form of web portal for easy and convenient retrieval of TSGs. Table 1 illustrates the comparison of our proposed BuffExDb with Si *et al*. [26] and Young *et al*. [25] based on the number of BioProjects, BioSample, RNA-Seq data, tissue, and user interface. BuffExDb is built to provide an expression profile of 76 tissues from 438 BioSamples. The user interface provides easy access and retrieval of the expression and TSG information. The tissues included in BuffExDb, which were not included in previous databases, are the cardiac atrium from the cardiovascular system; corpus luteum, granulosa cells, and female gonad from the female reproductive system; medulla oblongata, occipital cortex, and pineal gland from the central nervous system; white blood cells and palatine tonsil from the blood/immune system, rumen and reticulum from the alimentary canal; and others such as longissimus thoracis muscle, lung parenchyma, and ear skin. Tissue-specific biomarkers for these tissues can help in identifying diseases and traits controlled by these tissues. For instance, tissue-specific biomarkers for cardiac atrium may help in the identification of atrial fibrillation, which has been frequently observed in milking cows and consequently affects milk production [62]. The corpus luteum is involved in progesterone production and plays an important role in many reproductive processes, while the granulosa cells play a significant role in the growth and development of mammalian ovarian follicles. Granulosa cells can be used to study the adaptive response of buffaloes to heat [63]. Similarly, the pineal gland secretes and expresses specific proteins crucial for various physiological functions. Studies have noted that certain pineal proteins in buffaloes upregulate specific antioxidant defence mechanisms, offering potential utility in mitigating oxidative stress-induced neuronal disorders [64]. However, the fact cannot be overlooked that white blood cells and palatine tonsils play extremely important roles in the immune defence mechanism and protect the body from foreign molecule attack [65]. The corpus luteum serves as the principle reproductive gland responsible for progesterone production, essential for initiating and sustaining the gestation phase and successive implantation and embryonic development [66]. The additional, updated information on tissue-wise specific genes would be very beneficial for the bovine researchers and breeders in endeavour of trait and tissue-specific concerns of bovines.

**Table 1.** Comparison of BuffExDb with related databases

	BuffExDb	Si *et al*. [26]	Young *et al*. [25]
Buffalo species	Water and Swamp buffalo	Water and Swamp buffalo	Water buffalo
BioProjects	23	13	2
BioSample	438	355	220
RNA-Seq data	2429	355	2168
Tissue	76	50	57
User interface	Yes	No	No

### Utility of the Buffalo expression database, BuffExDb

The BuffExDb is a comprehensive collection of information on the transcriptomic profile of various tissues and cell types of the buffalo species. It includes expression information of both coding and noncoding transcripts from 63 tissues of water buffalo and 30 tissues of swamp buffalo, which is for the first time in a web-based form. The gene expression data for each tissue can be obtained in both FPKM and TPM and can be visualized using the heat map for a given range of FPKM values as provided by the user. This will help in identifying specific genes in a particular range of expression and observing the expression levels in different samples for that tissue. The expression values can be further utilized to identify differentially expressed genes between various biological conditions and tissues. The database also provides a gene-wise visualization of gene expression in each tissue enabling the user to identify the tissues in which the gene is highly or lowly expressed. The list of TSGs can be useful to understand various underlying molecular and biological mechanisms in each tissue and help in developing diagnostic and prognostic markers for various diseases and traits in the buffalo species. The database has also integrated functional annotation from KEGG and Reactome databases and annotations of the PANTHER classification system allowing users to explore the biological significance of their results. The user interface provides easy access to and interpretation of the expression profiles and TSGs in various tissues through user-friendly browsing, searching, and visualization of the expression data, with easy access to downloadable files for offline use.

## Conclusion

This study is comprehensive in cataloguing the globally scattered genomic information on buffalo gene expressions available to date. BuffExDb stands out as the first of its kind, offering two key features: an extensive collection of 2429 RNA-Seq datasets from 76 different tissues and cell types of buffalo and a web-based platform enabling users to retrieve TSG expression data, all annotated using the latest buffalo reference genome. It furnishes an extensive platform for delving into gene expression dynamics, regulatory pathways, and functional genomics within buffalo populations. The TSGs along with their functional annotation will provide insights into the molecular mechanisms involved in tissue-specific functions and diseases to enhance buffalo production potential. Tissue-specific biomarkers will also help in identifying the tissue–trait relation, which will, in turn, help in enhancing the traits for improving buffalo breeding, milk production, and reproductive efficiency. Researchers can explore gene expression across various body organs and tissues, from male and female samples, expanding different health conditions to gain an understanding of the biological interactions in this species. By leveraging the transcriptome database, BuffExDb, researchers can advance their understanding of buffalo biology and pave the way for precision breeding strategies and personalized approaches in buffalo breeding and conservation efforts.

## Supplementary Material

baae128_Supp

## Data Availability

The datasets presented in this study can be found in online repositories. The details of the repositories with their BioProject number(s) can be found in the Supplementary material as well as in our database, i.e. BuffExDb. The web/ftp address at which the database is available is http://46.202.167.198/buffex/.
